# Supplementary material for: Quantitative analysis of heparan sulfate using isotopically labeled calibrants
Source: Commun Biol. 2020 Aug 4;3:425. doi: 10.1038/s42003-020-01150-6 (PMC7403576; doi:10.1038/s42003-020-01150-6)
Supplement: Supplementary file 1 — Supplementary Information [file 42003_2020_1150_MOESM1_ESM.pdf]

Quantitative analysis of heparan sulfate using isotopically labeled calibrants

(Supporting information)

Supplementary Figures

Supplementary Fig. S1

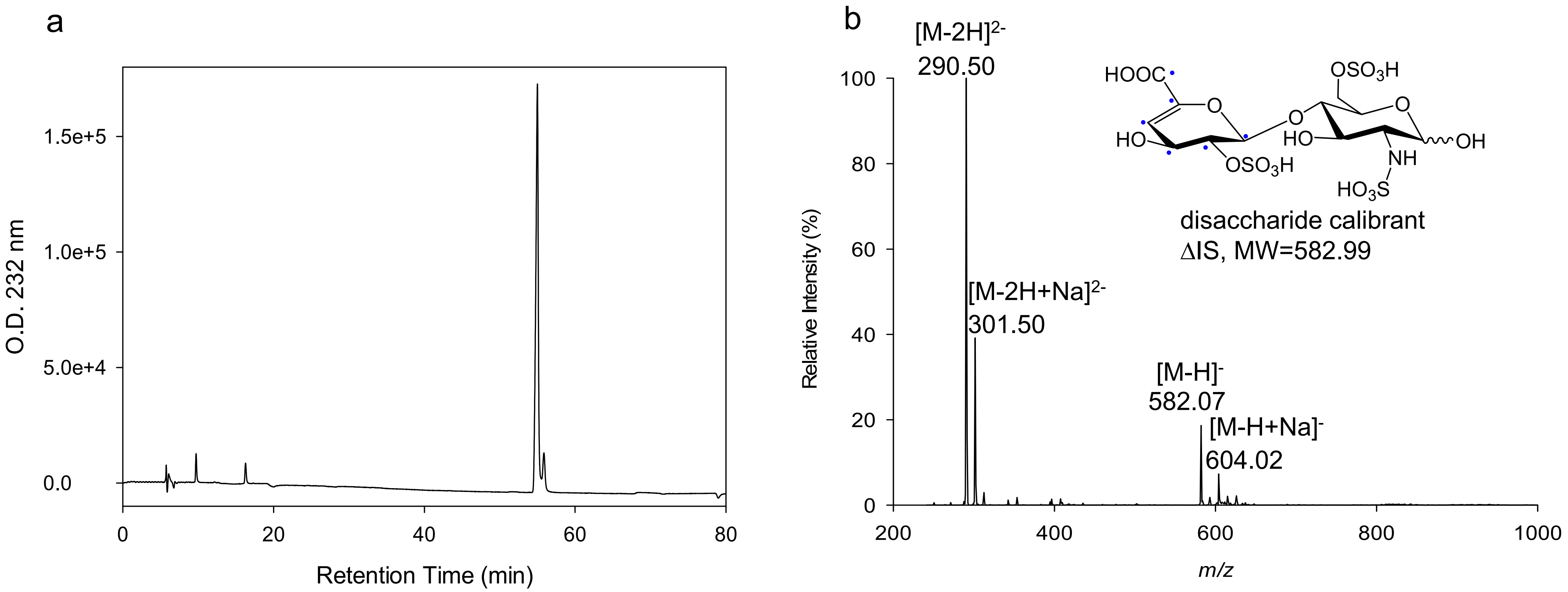

Supplementary Fig. 1. Purity analysis of disaccharide calibrant ΔIS. Panel **a** shows the SAX-HPLC chromatogram. A major single peak with a small shoulder peak was eluted at 56 min, suggesting that the chemical purity of disaccharide calibrant ΔIS is high. The small shoulder peak is an anomeric isomer. Panel **b** shows the ESI-MS spectrum of disaccharide calibrant ΔIS. The measured MW was 583.04, which is very close to the calculated value of 582.99. No signal in the m/z value of 287.48 $[M-2H]^{2-}$ and 575.97 $[M-H]^{-}$, the molecular ions representing unlabeled ΔIS counterpart. The data suggest that our preparation of disaccharide calibrant ΔIS standard has high isotopic purity.

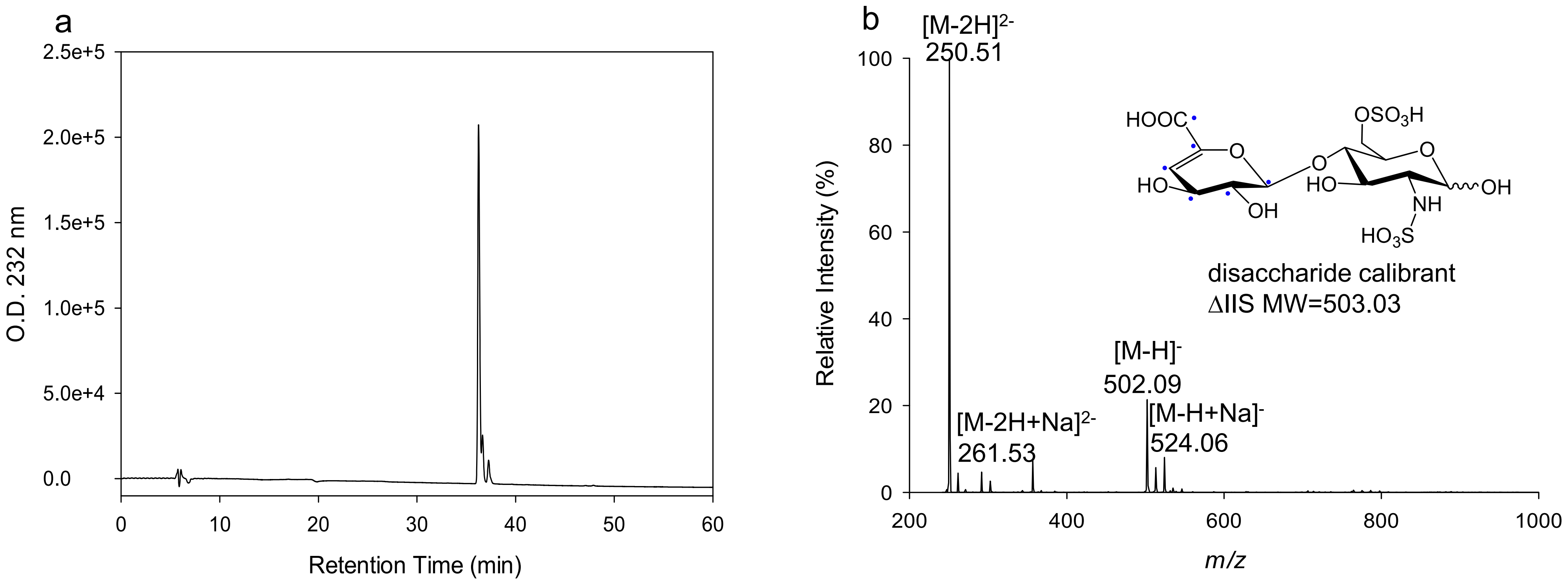

Supplementary Fig. 2. Purity analysis of disaccharide calibrant ΔIIS. Panel **a** shows the SAX-HPLC chromatogram. A major single peak with a small shoulder peak was eluted at 36 min, suggesting that the chemical purity of disaccharide calibrant ΔIIS is high. The small shoulder peak is an anomeric isomer. Panel **b** shows the ESI-MS spectrum of disaccharide calibrant ΔIIS. The measured MW was 503.04, which is very close to the calculated value of 503.03. No signal in the m/z value of 247.51 $[M-2H]^{2-}$ and 496.01 $[M-H]^{-}$, the molecular ions representing unlabeled ΔIIS counterpart. The data suggest that our preparation of disaccharide calibrant ΔIIS standard has high isotopic purity.

Supplementary Fig. S3

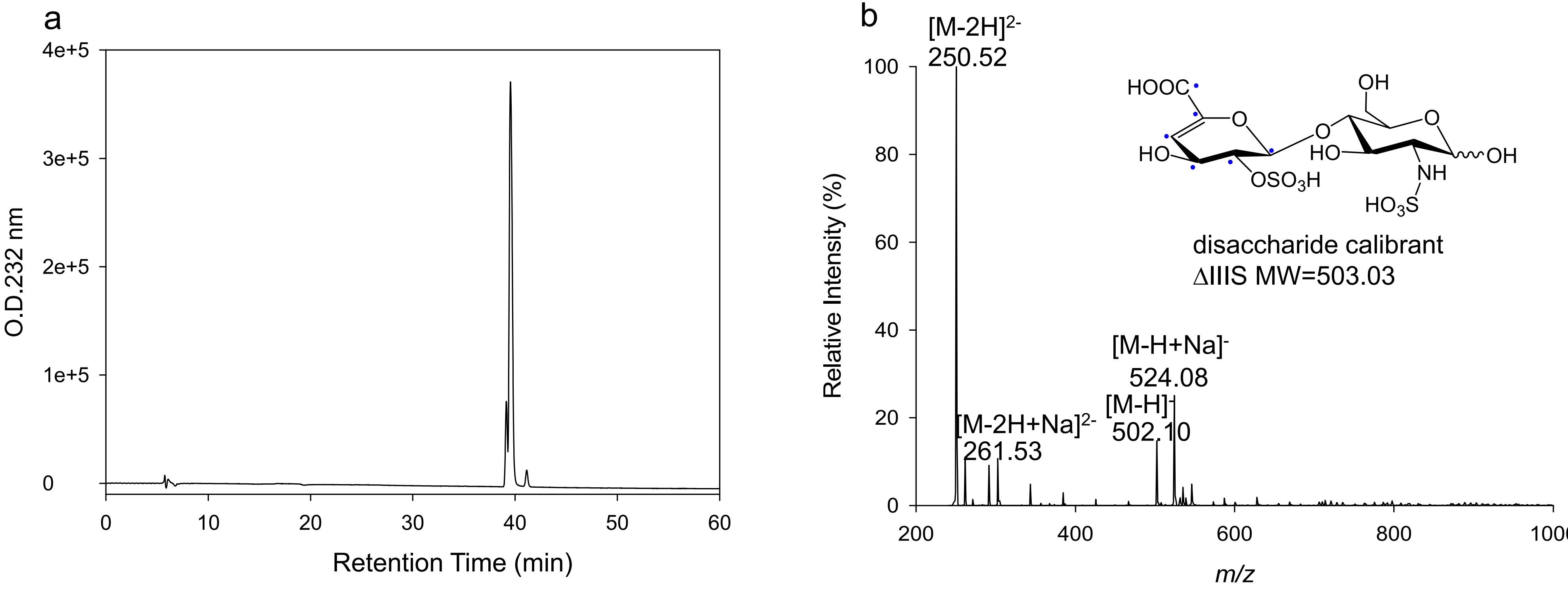

Supplementary Fig. 3. Purity analysis of disaccharide calibrant ΔIIIS. Panel **a** shows the SAX-HPLC chromatogram. A major single peak with a small shoulder peak was eluted at 39 min, suggesting that the chemical purity of disaccharide calibrant ΔIIIS is high. The small shoulder peak is an anomeric isomer. Panel **b** shows the ESI-MS spectrum of disaccharide calibrant ΔIIIS. The measured MW was 503.06, which is very close to the calculated value of 503.03. No signal in the m/z value of 247.51 $[M-2H]^{2-}$ and 496.01 $[M-H]^{-}$, the molecular ions representing unlabeled ΔIIIS counterpart. The data suggest that our preparation of disaccharide calibrant ΔIIIS standard has high isotopic purity.

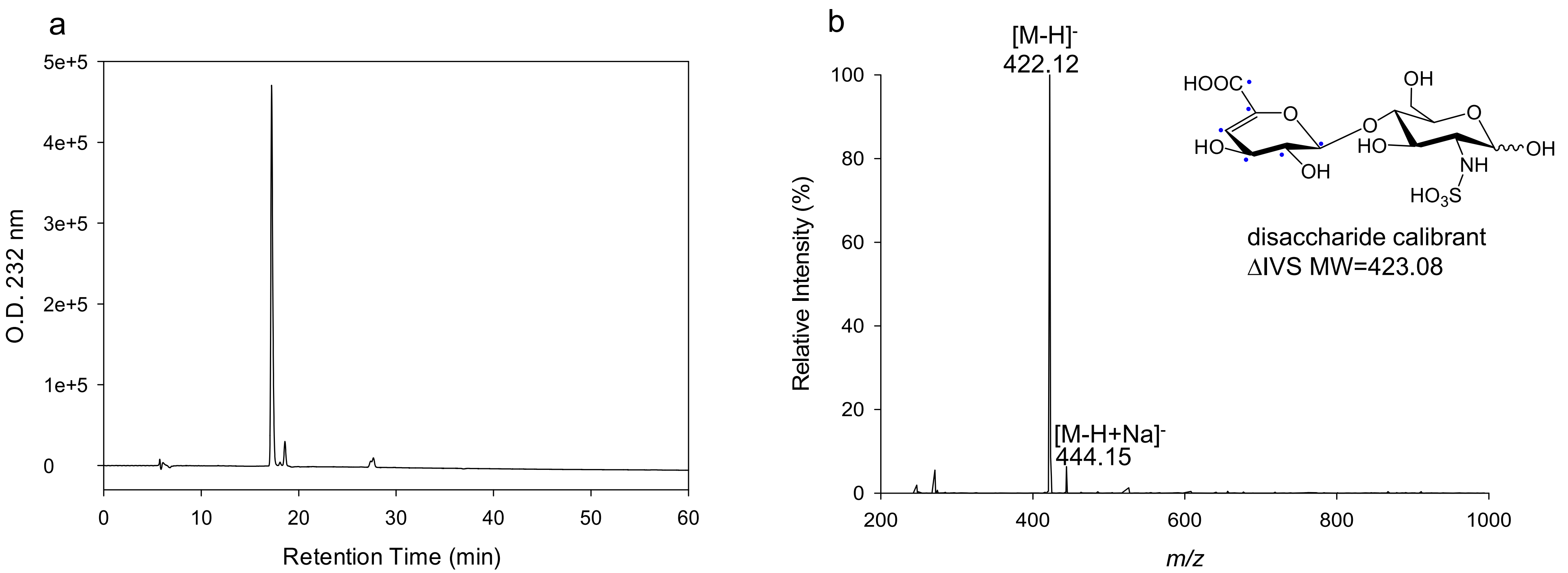

Supplementary Fig. 4. Purity analysis of disaccharide calibrant ΔIVS. Panel **a** shows the SAX-HPLC chromatogram. A major single peak was eluted at 17 min, suggesting that the chemical purity of disaccharide calibrant ΔIVS is high. Panel **b** shows the ESI-MS spectrum of disaccharide calibrant ΔIVS. The measured MW was 423.12, which is very close to the calculated value of 423.08. No signal in the m/z value of 416.06 $[M-H]^-$, the molecular ions representing unlabeled ΔIVS counterpart. The data suggest that our preparation of disaccharide calibrant ΔIVS standard has high isotopic purity.

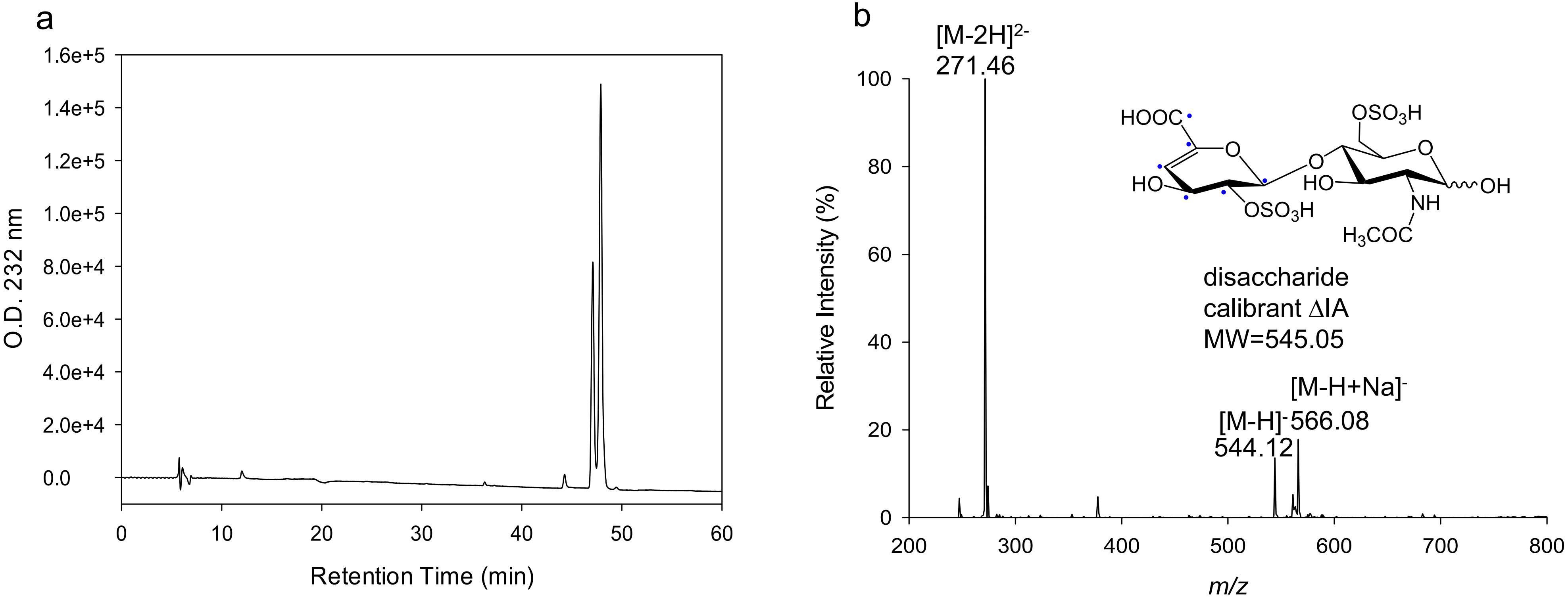

Supplementary Fig. 5. Purity analysis of disaccharide calibrant ΔIA. Panel **a** shows the SAX-HPLC chromatogram. A major single peak with a shoulder peak was eluted at 47 min, suggesting that the chemical purity of disaccharide calibrant ΔIA is high. The shoulder peak is an anomeric isomer. Panel **b** shows the ESI-MS spectrum of disaccharide calibrant ΔIA. The measured MW was 545.12, which is very close to the calculated value of 545.05. No signal in the m/z value of 268.51 $[M-2H]^{2-}$ and 538.03 $[M-H]^{-}$, the molecular ions representing unlabeled ΔIA counterpart. The data suggest that our preparation of disaccharide calibrant ΔIA standard has high isotopic purity.

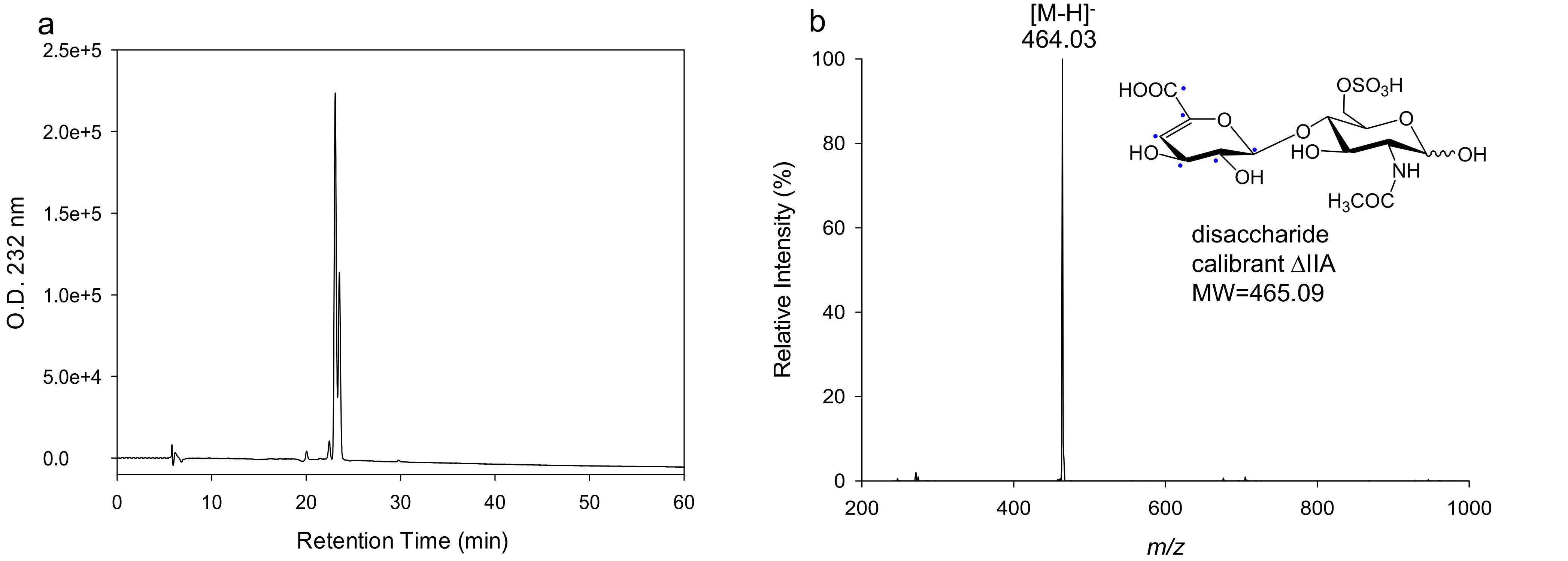

Supplementary Fig. 6. Purity analysis of disaccharide calibrant ΔIIA. Panel **a** shows the SAX-HPLC chromatogram. A major single peak with a shoulder peak was eluted at 24 min, suggesting that the chemical purity of disaccharide calibrant ΔIIA is high. The shoulder peak is an anomeric isomer. Panel **b** shows the ESI-MS spectrum of disaccharide calibrant ΔIIA. The measured MW was 465.03, which is very close to the calculated value of 465.09. No signal in the m/z value of 458.07 $[M-H]^-$, the molecular ions representing unlabeled ΔIIA counterpart. The data suggest that our preparation of disaccharide calibrant ΔIIA standard has high isotopic purity.

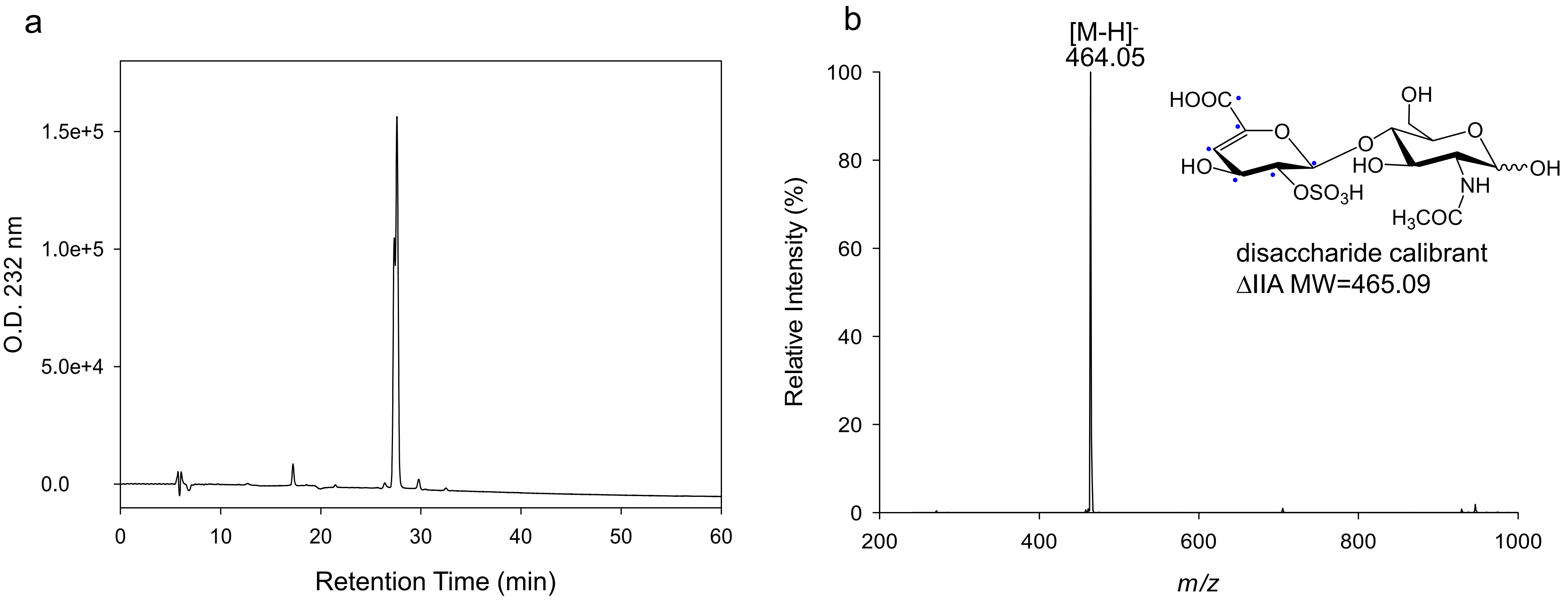

Supplementary Fig. 7. Purity analysis of disaccharide calibrant ΔIIIA. Panel **a** shows the SAX-HPLC chromatogram. A major single peak with a shoulder peak was eluted at 27 min, suggesting that the chemical purity of disaccharide calibrant ΔIIIA is high. The small shoulder peak is an anomeric isomer. Panel **b** shows the ESI-MS spectrum of disaccharide calibrant ΔIIIA. The measured MW was 465.05, which is very close to the calculated value of 465.09. No signal in the m/z value of 458.07 [M-H]$^-$, the molecular ions representing unlabeled ΔIIIA counterpart. The data suggest that our preparation of disaccharide calibrant ΔIIIA standard has high isotopic purity.

Supplementary Fig. S8

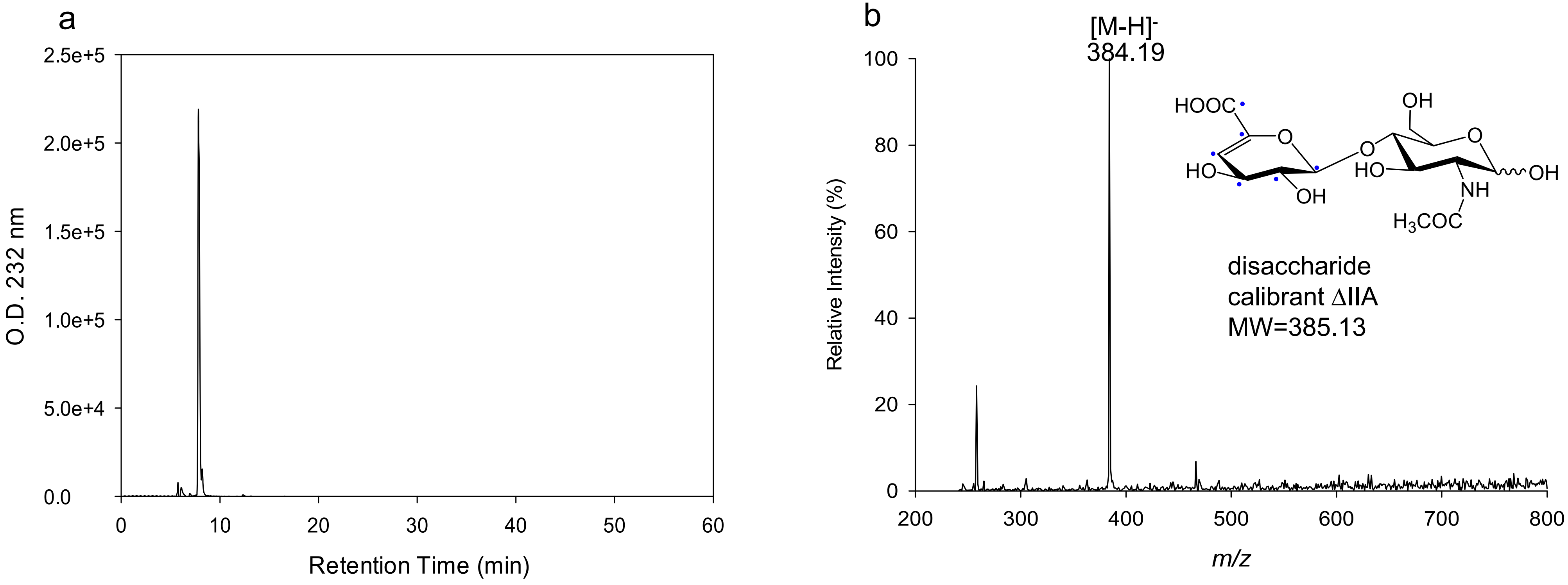

Supplementary Fig. 8. Purity analysis of disaccharide calibrant ΔIVA. Panel **a** shows the SAX-HPLC chromatogram. A major single peak was eluted at 8 min, suggesting that the chemical purity of disaccharide calibrant ΔIVA is high. Panel **b** shows the ESI-MS spectrum of disaccharide calibrant ΔIVA. The measured MW was 385.19, which is very close to the calculated value of 385.13. No signal in the m/z value of 378.11 $[M\text{-}H]^-$, the molecular ions representing unlabeled ΔIVA counterpart. The data suggest that our preparation of disaccharide calibrant ΔIVA standard has high isotopic purity.

Supplementary Fig S9

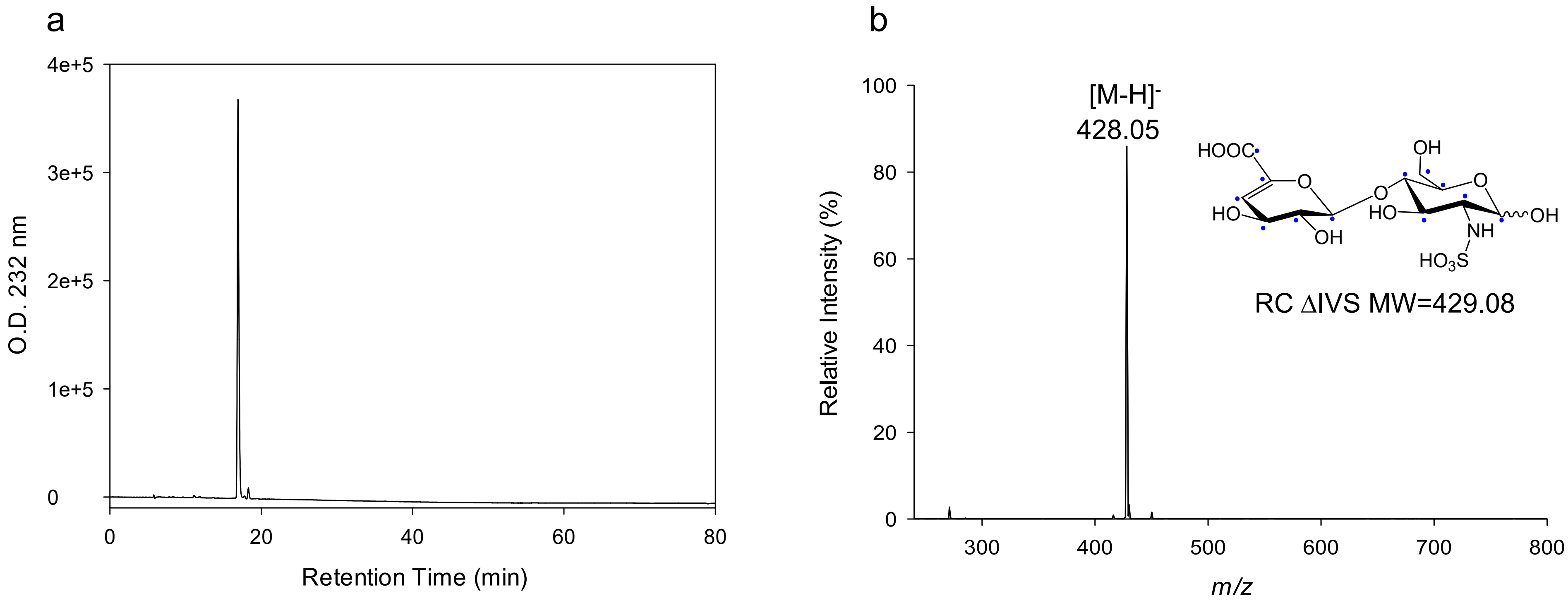

Supplementary Fig. 9. Disaccharide compositional analysis of RC standard. Panel **a** shows the SAX-HPLC chromatogram of RC standard after the digestion with heparin lyases. Only a single disaccharide of RC ΔIVS was observed, confirming that the RC standard is N-sulfo heparosan with the anticipated disaccharide repeating unit. Panel **b** shows the ESI-MS spectrum of the disaccharide from RC standard. The measured MW of the disaccharide was 429.05, which is close to the calculated value of 429.08. No signals at the range of m/z of 416.06 to 422.08 were observed, suggesting that the RC standard has high isotopic purity.

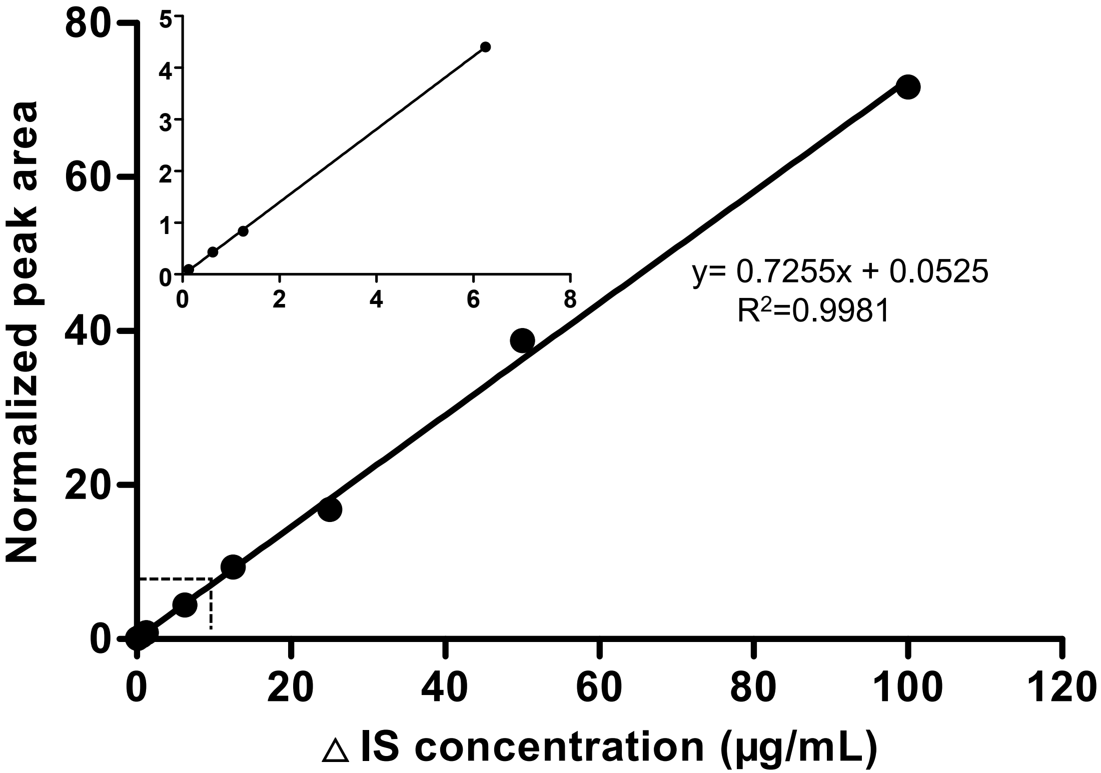

Supplementary Fig. 10. Linear dynamic range curve of ∆IS. The curve and linear equation of normalized peak area as a function of concentration for ∆IS are shown. The concentration of ∆IS used for LC-MS/MS analysis were 0.125, 0.625, 1.25, 6.25, 12.5, 25, 50 and 100 µg/mL, mixing with 1.25 µg/mL disaccharide calibrant ∆IS . Data represent means ± S.D. (n=3)

Supplementary Fig S11

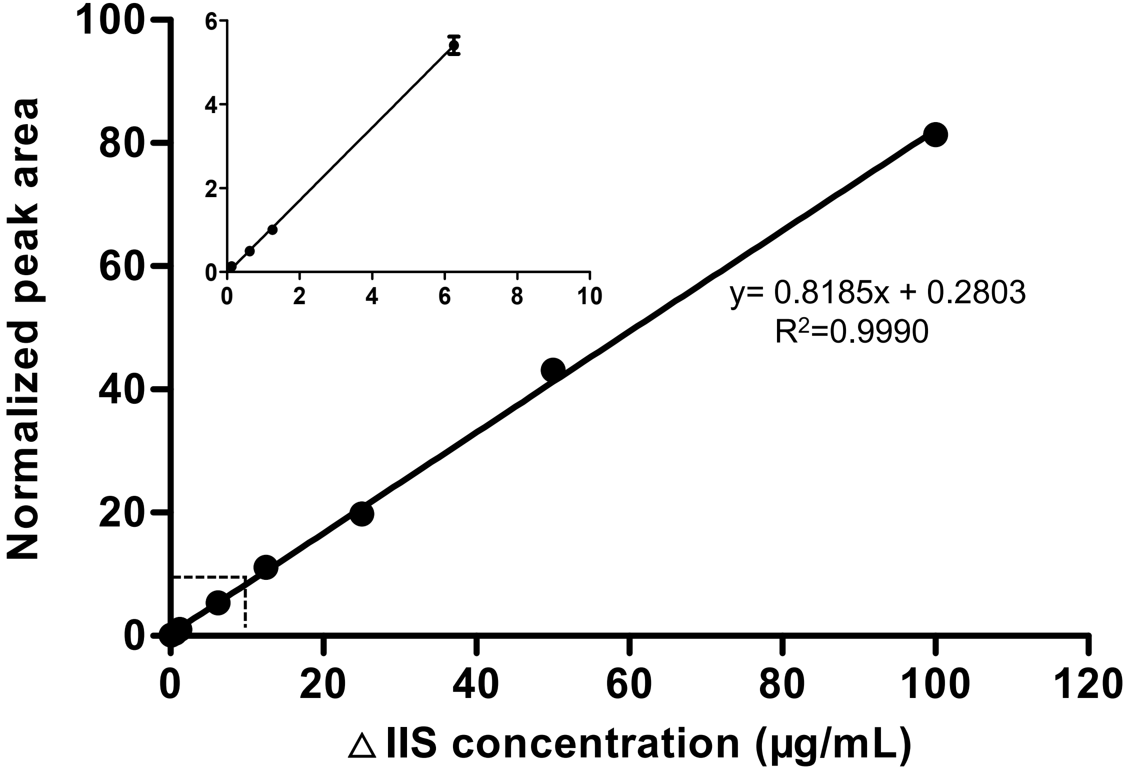

Supplementary Fig. 11. Linear dynamic range curve of ∆IIS. The curve and linear equation of normalized peak area as a function of concentration for ∆IIS are shown. The concentration of ∆IIS used for LC-MS/MS analysis were 0.125, 0.625, 1.25, 6.25, 12.5, 25, 50 and 100 μg/mL, mixing with 1.25 μg/mL disaccharide calibrant ∆IIS . Data represent means ± S.D. (n=3)

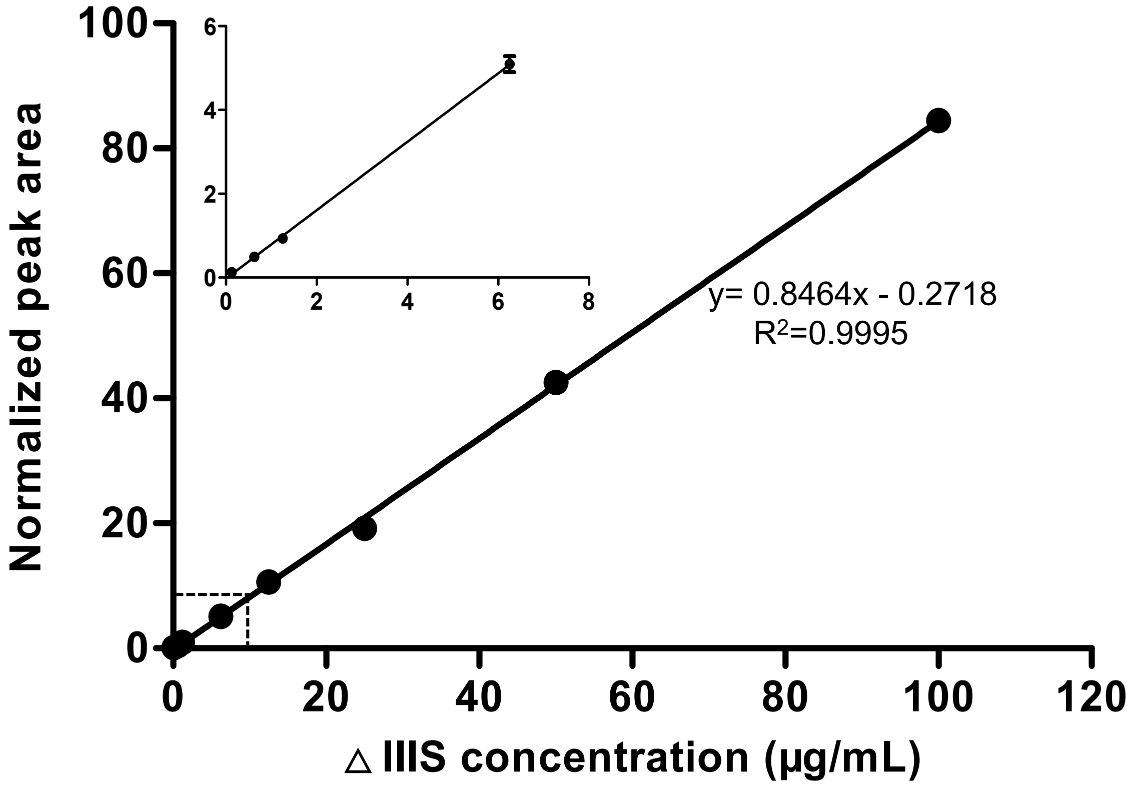

Supplementary Fig. 12. Linear dynamic range curve of ∆IIIS. The curve and linear equation of normalized peak area as a function of concentration for ∆IIIS are shown. The concentration of ∆IIIS used for LC-MS/MS analysis were 0.125, 0.625, 1.25, 6.25, 12.5, 25, 50 and 100 μg/mL, mixing with 1.25 μg/mL disaccharide calibrant ∆IIIS . Data represent means ± S.D. (n=3)

Supplementary Fig S13

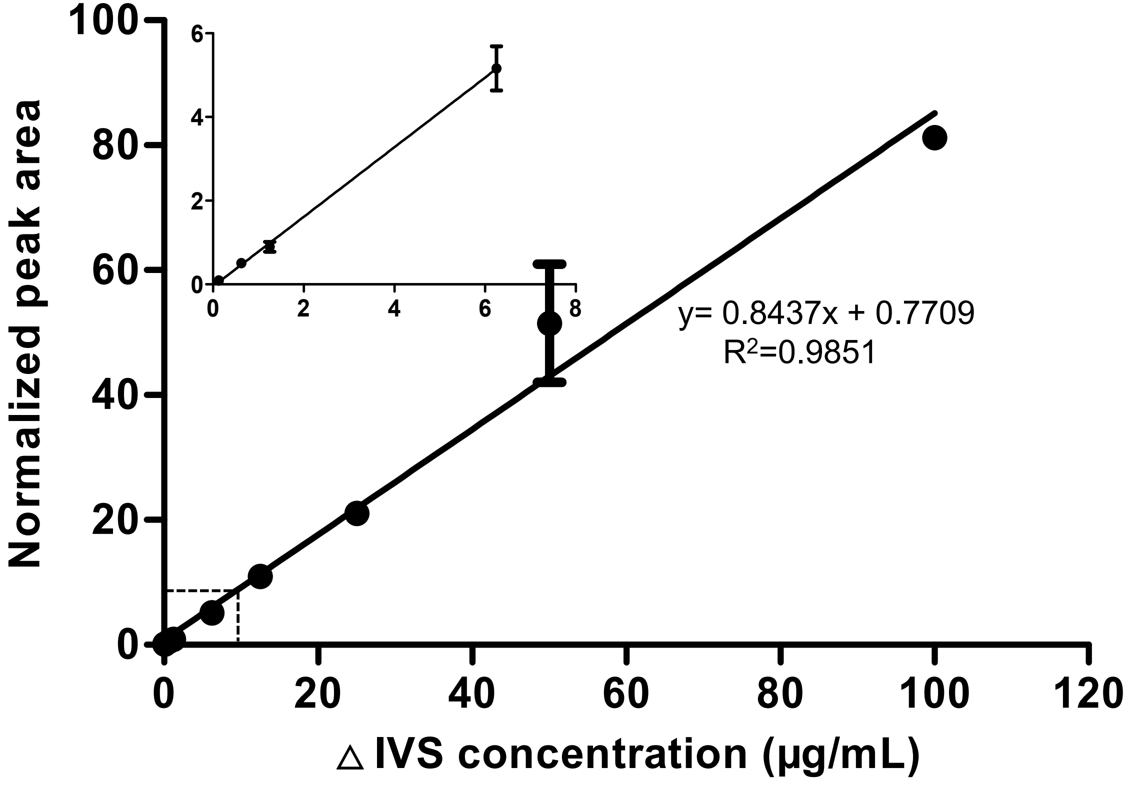

Supplementary Fig. 13. Linear dynamic range curve of ∆IVS. The curve and linear equation of normalized peak area as a function of concentration for ∆IVS are shown. The concentration of ∆IVS used for LC-MS/MS analysis were 0.125, 0.625, 1.25, 6.25, 12.5, 25, 50 and 100 µg/mL, mixing with 1.25 µg/mL disaccharide calibrant ∆IVS . Data represent means ± S.D. (n=3)

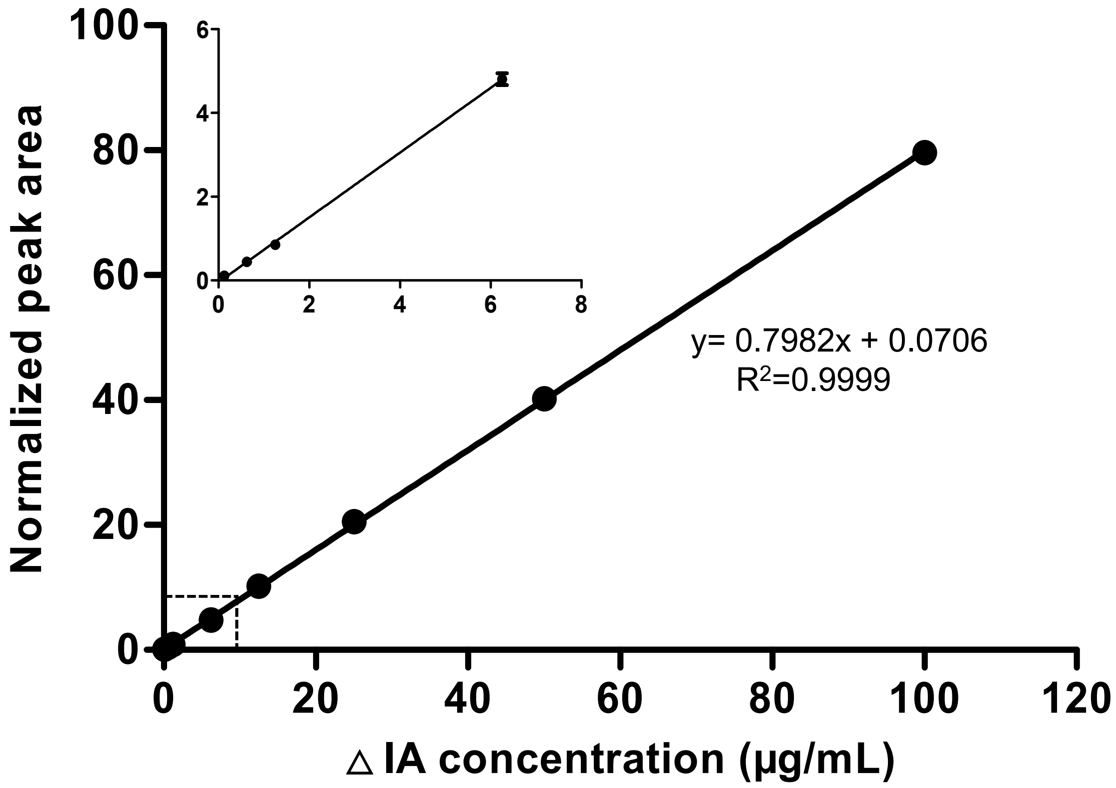

Supplementary Fig. 14. Linear dynamic range curve of ∆IA. The curve and linear equation of normalized peak area as a function of concentration for ∆IA are shown. The concentration of ∆IA used for LC-MS/MS analysis were 0.125, 0.625, 1.25, 6.25, 12.5, 25, 50 and 100 µg/mL, mixing with 1.25 µg/mL disaccharide calibrant ∆IA . Data represent means ± S.D. (n=3)

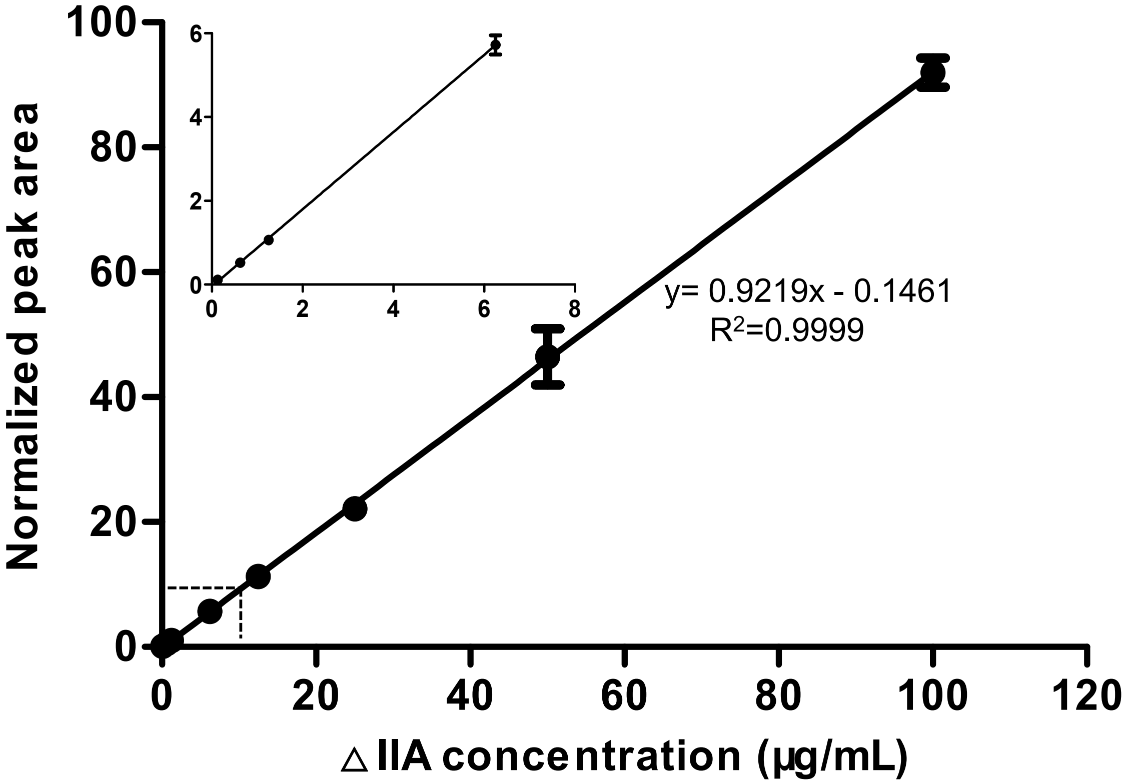

Supplementary Fig. 15. Linear dynamic range curve of ΔIIA. The curve and linear equation of normalized peak area as a function of concentration for ΔIIA are shown. The concentration of ΔIIA used for LC-MS/MS analysis were 0.125, 0.625, 1.25, 6.25, 12.5, 25, 50 and 100 μg/mL, mixing with 1.25 μg/mL disaccharide calibrant ΔIIA . Data represent means ± S.D. (n=3)

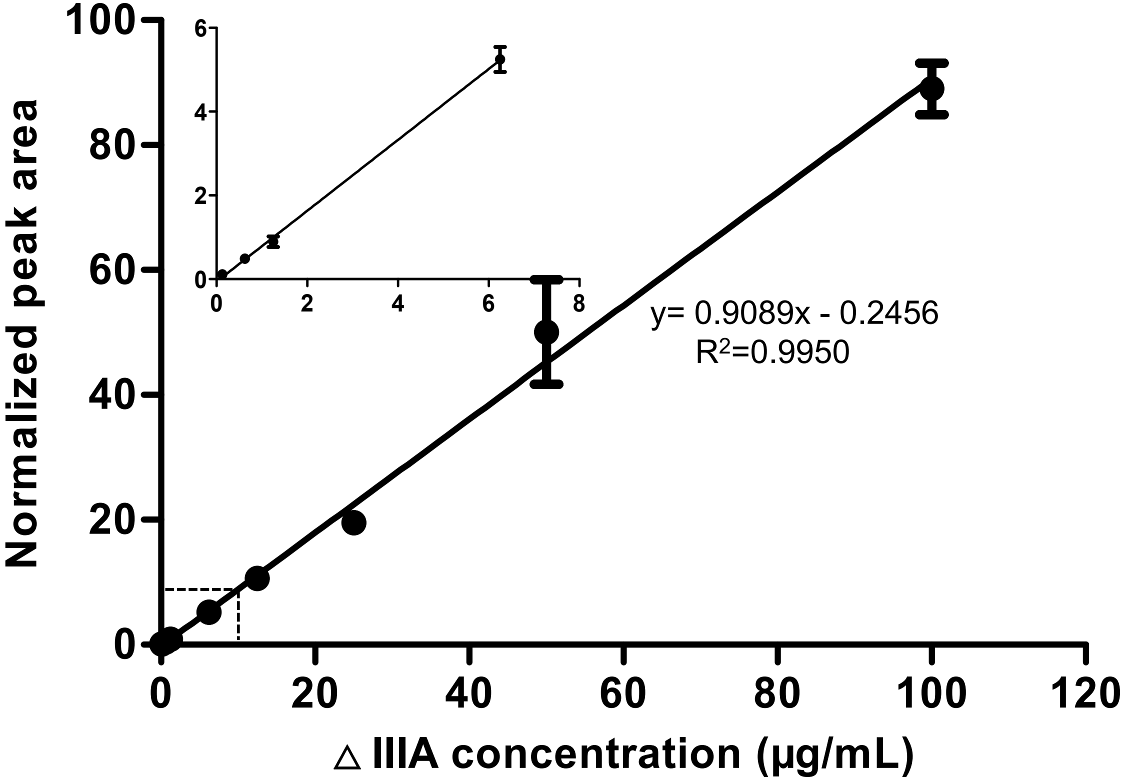

Supplementary Fig. 16. Linear dynamic range curve of ΔIIIA. The curve and linear equation of normalized peak area as a function of concentration for ΔIIIA are shown. The concentration of ΔIIIA used for LC-MS/MS analysis were 0.125, 0.625, 1.25, 6.25, 12.5, 25, 50 and 100 μg/mL, mixing with 1.25 μg/mL disaccharide calibrant ΔIIIA . Data represent means ± S.D. (n=3)

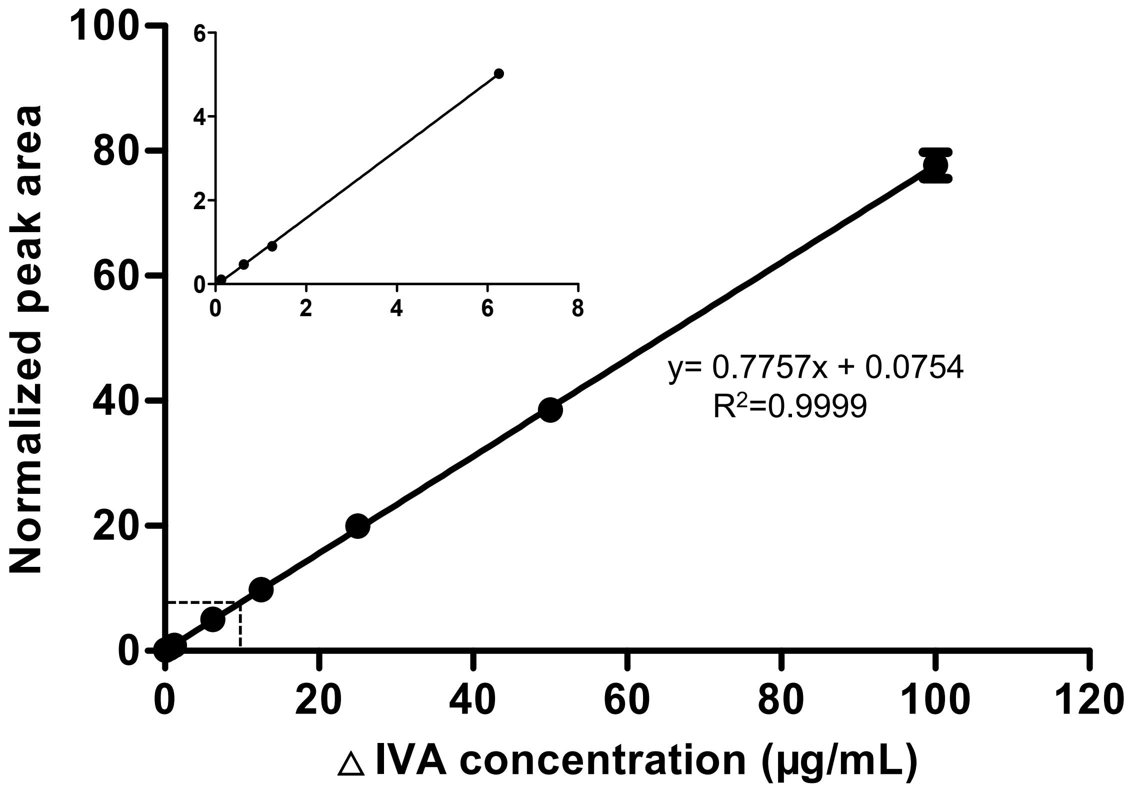

Supplementary Fig. 17. Linear dynamic range curve of ∆IVA. The curve and linear equation of normalized peak area as a function of concentration for ∆IVA are shown. The concentration of ∆IVA used for LC-MS/MS analysis were 0.125, 0.625, 1.25, 6.25, 12.5, 25, 50 and 100 μg/mL, mixing with 1.25 μg/mL disaccharide calibrant ∆IVA . Data represent means ± S.D. (n=3)

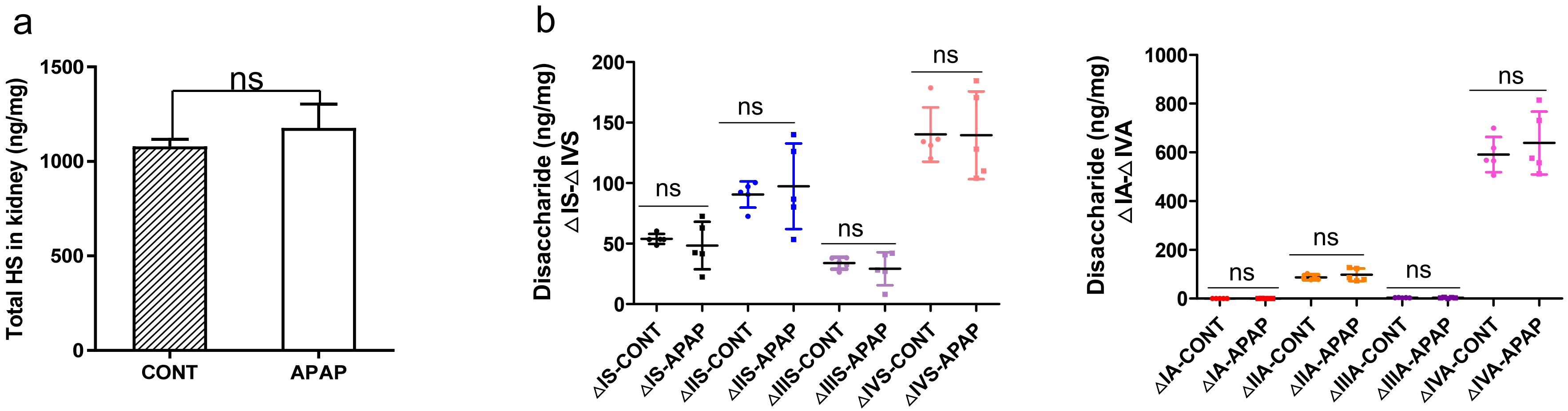

Supplementary Fig. 18. **Demonstrate of the utilities of quantitative LC-MS/MS method in mice kidney heparan sulfate.** Panel **a** shows the total amount heparan sulfate in the kidney of mice with or without APAP overdose. The data is presented as mean ± S.D. (n = 5). CONT represents the group of animals without APAP overdose; and APAP represents the group of animals with APAP overdose. The *p* value was determined by two-tailed unpaired t test, ns, not significant (p>0.05). Panel **b** shows the amount of individual disaccharides from mice kidney with or without APAP overdose.

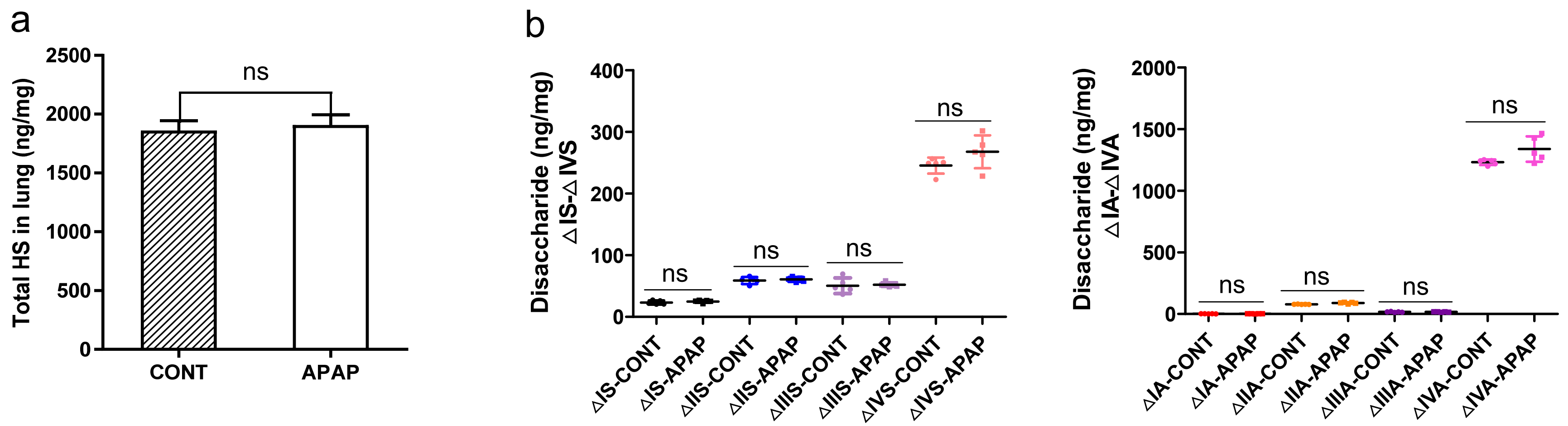

Supplementary Fig. 19. **Demonstrate of the utilities of quantitative LC-MS/MS method in mice lung heparan sulfate.** Panel **a** shows the total amount heparan sulfate in the lung of mice with or without APAP overdose. The data is presented as mean ± S.D. (n = 5). CONT represents the group of animals without APAP overdose; and APAP represents the group of animals with APAP overdose. The *p* value was determined by two-tailed unpaired t test, ns, not significant ($p>0.05$). Panel **b** shows the amount of individual disaccharides from mice lung with or without APAP overdose.

Supplementary Tables

Supplementary Table 1

The structure of ^{13}C-labeled oligosaccharides and prepared ^{13}C-labeled disaccharide calibrants, and corresponding theoretical molecular mass, amount and purity of ^{13}C-labeled disaccharide calibrants.

	^{13}C-labeled oligosaccharides	^{13}C-labeled disaccharide calibrants	Molecular mass	Amount (mg)	Purity (%)
Comp 1	GlcNS-GlcA*-GlcNS-IdoA2S*-	△UA2S*-GlcNS	503.03	2.0	97.3
	GlcNS-GlcA-pNP	△UA*-GlcNS	423.08	2.1	97.6
Comp 2	GlcNS6S-GlcA*-GlcNS6S-IdoA2S*-	△UA2S*-GlcNS6S	582.99	3.6	95.4
	GlcNS6S-GlcA-pNP	△UA*-GlcNS6S	503.03	10.0	95.2
Comp 3	GlcNS-GlcA-GlcNS-IdoA2S*-	△UA2S*-GlcNAc	465.09	2.7	96.5
	GlcNAc-GlcA*-GlcNAc-GlcA-pNP	△UA*-GlcNAc	385.13	1.3	99.6
Comp 4	GlcNS6S-GlcA-GlcNS6S-IdoA2S*-	△UA2S*-GlcNAc6S	545.05	3.0	97.3
	GlcNAc6S-GlcA*-GlcNAc6S-GlcA-pNP	△UA*-GlcNAc6S	465.09	2.2	95.4

*indicates the saccharide residue carries ^{13}C-labeled carbon atoms.

Supplementary Table 2

The ALT concentration in plasma 24 h after APAP administrations for mice liver tissue harvest

	ALT concentration (U/L)	
**Subject**	**Saline control mice**	**APAP-overdose mice**
1	37	1901
2	19	2658
3	23	3985
4	28	2684
5	27	1565
6	39	2483
7	23	2967
8	19	1830

Supplementary Table 3

The ALT concentration in plasma 24 h after APAP administrations for mice kidney and lung tissue harvest

	ALT concentration (U/L)	
**Subject**	**Saline control mice**	**APAP-overdose mice**
1	35.5	4710
2	13.5	3419
3	35	3264
4	23.5	3052
5	18	3366

Supplementary Table 4

Comparison of disaccharide composition of liver HS in the Saline control and APAP-overdose mice.

Subject	**Saline control mice liver- Disaccharides (ng/mg)**								**HS recovery yield**	**Total HS**
	ΔIS	ΔIIS	ΔIIIS	ΔIVS	ΔIA	ΔIIA	ΔIIIA	ΔIVA	**(%)**	**(ng/mg)**
1	16.7±0.5[a]	13.7±0.3	3.9±0.03	23.9±0.9	0.2±0.01	19.3±0.2	0.8±0.2	101.5±2.4	93.8±3.3[b]	191.4±1.7[c]
2	14.4±0.3	15.1±0.3	2.2±0.1	27.3±0.4	0.2±0.1	24.1±0.5	0.7±0.2	116.5±1.0	94.3±0.4	213.3±1.2
3	12.7±0.2	13.6±0.2	3.0±0.2	29.0±2.2	0.2±0.1	22.3±0.5	0.9±0.2	135.7±3.0	93.8±6.1	231.2±2.4
4	11.2±0.2	13.5±0.2	2.6±0.1	25.3±1.7	0.1±0.0	19.5±0.8	0.3±0.2	104.8±4.2	97.2±8.2	182.8±5.8
5	11.0±0.2	13.8±0.4	3.6±0.2	36.1±3.9	0.1±0.0	18.7±0.3	0.6±0.1	157.8±2.4	102.7±10.4	241.8±6.4
6	11.5±0.1	12.8±0.4	3.0±0.1	29.9±2.7	0.1±0.0	20.2±0.8	0.6±0.0	123.6±2.2	92.3±5.8	219.2±4.0
7	14.5±0.4	18.1±0.5	3.1±0.1	35.7±2.7	0.1±0.0	26.4±0.6	0.6±0.1	166.0±1.7	101.4±5.6	264.5±3.1
8	11.4±0.3	11.4±0.3	3.7±0.2	25.0±1.1	0.1±0.0	17.3±0.5	0.6±0.0	110.9±3.3	106.1±4.3	180.4±3.1
Ave (n=8)	12.9±2.1[d]	14.0±2.0	3.1±0.6	29.0±4.7	0.1±0.0	21.0±3.0	0.6±0.2	127.1±24.1	97.7±5.1[e]	215.6±29.8[f]
Subject	**APAP-overdose mice liver- Disaccharides (ng/mg)**								**HS recovery yield**	**Total HS**
	ΔIS	ΔIIS	ΔIIIS	ΔIVS	ΔIA	ΔIIA	ΔIIIA	ΔIVA	**(%)**	**(ng/mg)**
1	26.5±0.9	27.4±0.7	8.3±0.1	44.0±1.2	0.2±0.1	37.9±2.2	1.2±0.1	220.1±5.9	94.3±3.7	389.0±10.2
2	15.7±0.7	18.6±0.1	3.3±0.2	29.9±1.2	0.2±0.1	27.9±1.6	0.7±0.0	145.2±4.3	93.9±3.0	256.9±3.6
3	25.1±0.9	20.8±0.3	6.6±0.0	33.8±1.4	0.2±0.0	28.2±0.6	1.1±0.1	177.3±4.6	91.8±3.4	318.5±4.0
4	15.2±0.0	23.3±0.4	3.7±0.1	34.5±3.3	0.1±0.0	30.6±0.5	0.5±0.1	172.9±3.9	98.4±8.0	286.4±3.9
5	12.4±0.2	23.2±1.2	2.2±0.1	31.4±1.1	0.1±0.0	27.4±0.5	0.4±0.0	147.2±4.3	92.3±4.8	265.7±4.1
6	15.7±0.7	24.0±0.8	3.0±0.2	37.3±0.7	0.1±0.0	32.9±0.6	0.5±0.0	187.9±4.5	86.1±0.7	350.5±6.8
7	17.1±0.2	26.1±1.2	3.1±0.3	37.4±1.4	0.1±0.0	32.5±0.5	0.4±0.1	171.8±7.4	118.2±4.4	244.5±7.9
8	12.3±0.5	21.2±0.3	2.1±0.1	30.2±2.8	0.1±0.0	27.1±1.1	0.3±0.1	149.7±5.0	102.9±9.3	214.8±8.4
Ave (n=8)	17.5±5.4	23.1±2.9	4.0±2.2	34.8±4.7	0.2±0.1	30.5±3.7	0.6±0.3	171.5±25.1	97.2±9.8	294.3±53.6

a, b and c indicate that the values were means of three measurements ± standard deviations. d, e and f present that the values were means of the measurements of eight mouse liver (n=8) ± standard deviations.

Supplementary Table 5

Comparison of disaccharide composition of kidney HS in the Saline control and APAP-overdose mice.

Subject	Saline control mice kidney- Disaccharides (ng/mg)								HS recovery yield (%)	Total HS (ng/mg)
	ΔIS	ΔIIS	ΔIIIS	ΔIVS	ΔIA	ΔIIA	ΔIIIA	ΔIVA		
1	53.4±5.1	90.6±6.7	32.1±1.6	131.2±5.7	0.6±0.0	85.5±0.5	3.6±0.4	567.8±7.7	87.9±5.0	1096.4±29.9
2	60.5±5.7	100.4±6.5	38.3±2.0	178.7±7.0	0.7±0.2	103.2±5.8	4.7±0.3	699.5±8.4	109.7±4.2	1079.2±7.9
3	53.6±2.4	72.5±4.0	26.4±0.5	136.3±16.0	0.4±0.1	78.3±4.0	2.9±0.2	565.1±31.8	97.2±9.6	964.5±20.2
4	53.5±6.7	92.4±8.4	34.8±1.5	120.5±4.8	0.6±0.1	78.7±2.5	3.1±0.2	506.4±12.8	83.8±0.5	1059.6±27.8
5	48.7±1.7	97.2±3.9	37.9±2.7	134.3±9.2	0.6±0.1	91.5±1.6	3.5±0.2	618.0±10.0	85.9±5.4	1199.6±15.2
Ave (n=5)	53.9±4.2	90.6±10.8	33.9±5.0	140.2±22.4	0.6±0.1	87.4±10.3	3.6±0.7	591.4±72.2	92.9±10.7	1079.9±84.1
Subject	**APAP-overdose mice kidney- Disaccharides (ng/mg)**								**HS recovery yield (%)**	**Total HS (ng/mg)**
	ΔIS	ΔIIS	ΔIIIS	ΔIVS	ΔIA	ΔIIA	ΔIIIA	ΔIVA		
1	72.5±3.6	140.1±5.7	42.1±1.9	184.6±19.7	0.8±0.1	123.9±7.4	5.7±0.7	731.3±21.5	92.2±11.0	1414.2±6.5
2	22.3±1.0	53.5±1.7	8.1±0.1	104.1±0.6	0.1±0.0	72.7±1.6	0.2±0.0	557.2±11.7	85.3±0.1	962.7±17.1
3	41.5±0.7	86.7±1.9	27.0±0.6	128.1±13.8	0.7±0.1	86.2±2.6	3.1±0.3	576.6±12.3	87.1±9.3	1091.9±4.3
4	42.6±1.9	80.3±2.2	28.3±0.3	110.1±4.2	0.5±0.0	78.7±1.2	3.1±0.4	512.2±2.8	96.5±1.2	882.3±7.3
5	63.0±0.8	126.3±3.9	40.7±0.6	170.7±6.6	0.8±0.1	126.8±3.8	4.6±0.5	814.6±7.5	87.5±2.7	1531.2±19.5
Ave (n=5)	48.4±19.7	97.4±35.3	29.2±13.7	139.5±36.2	0.6±0.3	97.7±25.7	3.3±2.1	638.4±128.5	89.7±4.6	1176.4±283.6

Supplementary Table 6

Comparison of disaccharide composition of lung HS in the Saline control and APAP-overdose mice.

Subject	Saline control mice lung- Disaccharides (ng/mg)								HS recovery yield (%)	Total HS (ng/mg)
	ΔIS	ΔIIS	ΔIIIS	ΔIVS	ΔIA	ΔIIA	ΔIIIA	ΔIVA		
1	27.3±2.7	65.9±2.1	69.6±1.4	250.5±8.8	1.9±0.2	80.8±2.1	21.6±2.7	1243.0±6.7	90.7±2.2	1096.4±29.9
2	25.9±1.2	59.7±1.5	55.1±1.5	249.1±15.2	1.8±0.4	78.9±1.7	18.1±2.1	1199.8±31.2	91.2±6.4	1079.2±7.9
3	20.7±0.7	58.1±0.6	47.0±0.5	256.5±31.7	1.8±0.2	78.7±3.0	17.2±0.5	1245.5±61.8	97.3±13.7	964.5±20.2
4	20.6±0.8	50.4±2.0	44.4±1.3	223.0±9.4	1.5±0.3	76.8±3.6	14.6±0.5	1252.5±13.1	85.7±4.1	1059.6±27.8
5	22.0±1.6	60.5±1.3	36.4±0.7	249.0±19.4	1.6±0.2	78.0±1.3	12.2±0.9	1218.6±33.3	94.2±5.4	1199.6±15.2
Ave (n=5)	23.3±3.1	58.9±5.6	50.5±12.6	245.6±13.0	1.7±0.1	78.6±1.5	16.7±3.6	1231.9±22.0	91.8±4.3	1862.5±82.6
Subject	APAP-overdose mice lung- Disaccharides (ng/mg)								HS recovery yield (%)	Total HS (ng/mg)
	ΔIS	ΔIIS	ΔIIIS	ΔIVS	ΔIA	ΔIIA	ΔIIIA	ΔIVA		
1	25.6±0.2	65.6±2.1	49.7±0.9	301.7±33.2	2.0±0.1	93.7±2.7	15.4±0.8	1465.9±50.8	101.6±11.0	1414.2±6.5
2	26.6±1.4	61.2±1.8	51.6±1.6	263.3±18.5	2.0±0.2	87.0±7.1	18.6±1.3	1272.8±10.9	102.8±8.8	962.7±17.1
3	25.0±0.5	57.9±2.3	53.1±0.6	278.7±12.5	1.9±0.2	95.6±8.0	19.5±1.3	1427.1±26.4	102.8±4.4	1091.9±4.3
4	26.7±0.9	62.8±0.7	58.3±1.4	267.4±7.5	1.9±0.2	90.6±7.7	18.7±1.4	1305.1±28.1	96.8±4.4	882.3±7.3
5	21.1±0.3	55.9±2.2	48.9±1.0	228.4±19.6	1.6±0.1	80.9±2.4	15.0±1.8	1223.1±58.9	89.3±8.0	1531.2±19.5
Ave (n=5)	25.0±2.3	60.7±3.9	52.3±3.7	267.9±26.7	1.9±0.1	89.5±5.9	17.4±2.1	1338.8±103.5	98.7±5.8	1906.3±89.0
