# Supplementary material for: Quantitative analysis of heparan sulfate using isotopically labeled calibrants
Source: Commun Biol. 2020 Aug 4;3:425. doi: 10.1038/s42003-020-01150-6 (PMC7403576; doi:10.1038/s42003-020-01150-6)
Supplement: Supplementary file 3 — Description of Additional Supplementary Files [file 42003_2020_1150_MOESM3_ESM.pdf]

Description of Additional Supplementary Files

File Name: **Supplementary Data 1**
Description: All the original data that were used to make figures presented in the manuscript
