# Supplementary material for: Quantitative analysis of heparan sulfate using isotopically labeled calibrants
Source: Commun Biol. 2020 Aug 4;3:425. doi: 10.1038/s42003-020-01150-6 (PMC7403576; doi:10.1038/s42003-020-01150-6)
Supplement: Supplementary file 5 — Reporting Summary [file 42003_2020_1150_MOESM5_ESM.pdf]

nature research

Corresponding author(s): Jian Liu

Last updated by author(s): Jun 25, 2020

Reporting Summary

Nature Research wishes to improve the reproducibility of the work that we publish. This form provides structure for consistency and transparency in reporting. For further information on Nature Research policies, see Authors & Referees and the Editorial Policy Checklist.

Statistics

For all statistical analyses, confirm that the following items are present in the figure legend, table legend, main text, or Methods section.

n/a	Confirmed	
☐	☒	The exact sample size (*n*) for each experimental group/condition, given as a discrete number and unit of measurement
☐	☒	A statement on whether measurements were taken from distinct samples or whether the same sample was measured repeatedly
☐	☒	The statistical test(s) used AND whether they are one- or two-sided<br>*Only common tests should be described solely by name; describe more complex techniques in the Methods section.*
☒	☐	A description of all covariates tested
☒	☐	A description of any assumptions or corrections, such as tests of normality and adjustment for multiple comparisons
☐	☒	A full description of the statistical parameters including central tendency (e.g. means) or other basic estimates (e.g. regression coefficient) AND variation (e.g. standard deviation) or associated estimates of uncertainty (e.g. confidence intervals)
☐	☒	For null hypothesis testing, the test statistic (e.g. F, t, r) with confidence intervals, effect sizes, degrees of freedom and P value noted<br>*Give P values as exact values whenever suitable.*
☒	☐	For Bayesian analysis, information on the choice of priors and Markov chain Monte Carlo settings
☒	☐	For hierarchical and complex designs, identification of the appropriate level for tests and full reporting of outcomes
☒	☐	Estimates of effect sizes (e.g. Cohen's d, Pearson's r), indicating how they were calculated

Our web collection on statistics for biologists contains articles on many of the points above.

Software and code

Policy information about availability of computer code

Data collection	TraceFinder 4.1 General Quan software (Thermo Fisher Scientific)
Data analysis	TraceFinder 4.1 General Quan software (Thermo Fisher Scientific)<br>GraphPad Prism 5

For manuscripts utilizing custom algorithms or software that are central to the research but not yet described in published literature, software must be made available to editors/reviewers. We strongly encourage code deposition in a community repository (e.g. GitHub). See the Nature Research guidelines for submitting code & software for further information.

Data

Policy information about availability of data

All manuscripts must include a data availability statement. This statement should provide the following information, where applicable:

- Accession codes, unique identifiers, or web links for publicly available datasets
- A list of figures that have associated raw data
- A description of any restrictions on data availability

All the mass spectral data related to this research were deposited in the MassIVE [doi:10.25345/C5F419]. Source data are available as Supplementary Data. The relevant data would be available from the corresponding author upon reasonable request

Field-specific reporting

Please select the one below that is the best fit for your research. If you are not sure, read the appropriate sections before making your selection.

☒ Life sciences ☐ Behavioural & social sciences ☐ Ecological, evolutionary & environmental sciences

For a reference copy of the document with all sections, see nature.com/documents/nr-reporting-summary-flat.pdf

Life sciences study design

All studies must disclose on these points even when the disclosure is negative.

Sample size	For the determination of HS amount in mice liver, sample size (n=8) was used and the p value was determined by unpaired t test using GraphPad Prism 5 software. For the determination of HS amount in mice lung and kidney, sample size (n=5) was used and the p value was determined by unpaired t test using GraphPad Prism 5 software.
Data exclusions	No
Replication	The quantitative analysis of HS in the mice liver was performed by comparing 8 control mice and 8 APAP-injured mice. The quantitative analysis of HS in the mice lung and kidney was performed by comparing 5 control mice and 5 APAP-injured mice. All samples were injected three times and the values were means of three measurements ± standard deviations. The statistical analysis of HS amount in liver (n=8), lung (n=5) and kidney (n=5) was achieved by p values determined by unpaired t test.
Randomization	Mice were randomly selected to be in either the APAP overdose or saline control group.
Blinding	The animal experiment was not blinded.

Reporting for specific materials, systems and methods

We require information from authors about some types of materials, experimental systems and methods used in many studies. Here, indicate whether each material, system or method listed is relevant to your study. If you are not sure if a list item applies to your research, read the appropriate section before selecting a response.

Materials & experimental systems

n/a	Involved in the study	
☒	☐	Antibodies
☒	☐	Eukaryotic cell lines
☒	☐	Palaeontology
☐	☒	Animals and other organisms
☒	☐	Human research participants
☒	☐	Clinical data

Methods

n/a	Involved in the study	
☒	☐	ChIP-seq
☒	☐	Flow cytometry
☒	☐	MRI-based neuroimaging

Animals and other organisms

Policy information about studies involving animals; ARRIVE guidelines recommended for reporting animal research

Laboratory animals	Male C57BL/6J 10 week old mice were used in this paper
Wild animals	N/A.
Field-collected samples	N/A.
Ethics oversight	All animal experiments were approved by the Institutional Animal Care and Use Committee (IACUC) of University of North Carolina at Chapel Hill (Chapel Hill, NC).

Note that full information on the approval of the study protocol must also be provided in the manuscript.
